# Attitude Synchronization of a Group of Rigid Bodies Using Exponential Coordinates

**DOI:** 10.3390/e25060832

**Published:** 2023-05-23

**Authors:** Miguel Sidón-Ayala, Javier Pliego-Jiménez, César Cruz-Hernandez

**Affiliations:** 1 Departamento de Electrónica y Telecomunicaciones, División de Física Aplicada, Centro de Investigación Científica y de Educación Superior de Ensenada, Carretera Ensenada-Tijuana 3918, Ensenada 22860, Mexico; miguels@cicese.edu.mx (M.S.-A.); jpliego@cicese.mx (J.P.-J.); 2 Programa Investigadores por México, Consejo Nacional de Humanidades Ciencias y Tecnologías, Av. Insurgentes Sur 1582, Mexico City 03940, Mexico

**Keywords:** synchronization, attitude control, multi-agent systems, spacecraft, exponential coordinates

## Abstract

Currently, managing a group of satellites or robot manipulators requires coordinating their motion and work in a cooperative way to complete complex tasks. The attitude motion coordination and synchronization problems are challenging since attitude motion evolves in non-Euclidean spaces. Moreover, the equation of motions of the rigid body are highly nonlinear. This paper studies the attitude synchronization problem of a group of fully actuated rigid bodies over a directed communication topology. To design the synchronization control law, we exploit the cascade structure of the rigid body’s kinematic and dynamic models. First, we propose a kinematic control law that induces attitude synchronization. As a second step, an angular velocity-tracking control law is designed for the dynamic subsystem. We use the exponential coordinates of rotation to describe the body’s attitude. Such coordinates are a natural and minimal parametrization of rotation matrices which almost describe every rotation on the Special Orthogonal group SO(3). We provide simulation results to show the performance of the proposed synchronization controller.

## 1. Introduction

Attitude stabilization and tracking are important objectives in automatic control [1] and robotics, since these problems appear in several tasks, such as aerospace tasks, robot force control, dexterous robot manipulation, assembly tasks, and vehicle orientation [2,3,4,5]. Compared to position trajectory tracking or regulation problems, attitude control is more involved. This is due to the attitude kinematics, and dynamics are nonlinear and evolve in non-Euclidian spaces such as the Special Orthogonal group SO(n) or the *n*-Sphere $S^{n}$ [6,7].

On the other hand, the coordination of multiple robots or spacecraft has received much attention in recent decades. Nowadays, robotic tasks are more demanding and challenging, requiring multiple robots to perform the task correctly. Cooperative robots take inspiration from the collective behaviors observed in nature, for instance, flocking birds or schooling fish [8,9]. Interesting collective behaviors in robotics include synchronization, flocking, formation, and consensus [10,11,12].

Attitude synchronization is fundamental in spacecrafts swarms. The formation of sensor arrays can expand the range of the system, as in deep-space interferometry [13,14], vision systems [15] and antenna arrays [16], to mention a few. Different approaches are used to represent the attitude of a rigid body, often trying to avoid the singularities produced by the Euler’s angle representation. As an example, Sharma and Kar [17] propose an almost global controller in the tangent bundle TSO(3) in order to achieve consensus in a group of rigid bodies under a directed graph communication. In this case, the attitude of the bodies is represented by means of rotation matrices. A quaternion-based synchronization controller for multiple spacecrafts is presented in [18]. The synchronization control law does not depend on the dynamic model of the space craft; however, it only works for a bidirectional ring topology. A novel control strategy for robust global attitude synchronization based on hybrid control theory is proposed in [19]. In this work, the attitude of the rigid body is described by the unit quaternion, and the hybrid controllers avoid the well-known unwinding phenomena. The problem of attitude consensus and velocity synchronization for a multi-agent rigid body system is addressed in [20]. The authors propose nonlinear control laws based on rotation matrices that achieve asymptotic consensus and synchronization. Nevertheless, the controller is limited for undirected graphs. An energy-shaping approach for attitude synchronization is proposed in [21]. The proposed control law works for directed and time-varying connected graphs.

On the other hand, Liu et al. [22] achieved attitude synchronization of spacecraft without needing velocity measurements, which is due to the representation of the rigid bodies by means of modified Rodrigues parameters (MRPs). Furthermore, Abdessameud et al. [10] employed the unit quaternion representation of the attitude of a spacecraft to cope with the attitude synchronization of multiple spacecraft under communication delays. In this case, the authors proposed a leader–follower scheme and also a graph-based scheme to solve the problem. Ref. Liu and Huang [23] also consider the leader–follower synchronization problem of a group of rigid bodies but under switching topologies. To solve this problem, the authors propose a distributed observer in combination with a distributed control law based on unit quaternion representation. Using the modified Rodrigues parameters, Ren [24] proposes two distributed control laws based on the passivity framework that achieves attitude synchronization. On the other hand, Zou [25] addressed the problem of attitude synchronization of rigid bodies when the reference attitude is only available for a few members of the team. The author proposed a discontinuous control law combined with a sliding mode observer.

The main contribution of this paper is the design of a distributed attitude synchronization control law over directed communication topologies for a group of fully actuated rigid bodies. The synchronization controller is obtained exploiting the cascade structure of the kinematic and dynamic model of the rigid body. In this regard, we propose two control loops. First, a kinematic control law (outer-loop controller) based on the exponential coordinates of rotation was design to achieve attitude synchronization. Then, an angular velocity tracking control (inner-loop controller) is proposed for the dynamic model. The stability of the closed-loop equilibrium point was proved by a strict Lyapunov function. The rest of the paper is organized as follows. In Section 2, the kinematic and dynamic models of rigid spacecrafts are presented as well as some key features of graph theory. Section 3 describes the proposed attitude consensus control law for the multi-agent system. Simulation results are reported in Section 4 and, lastly, some conclusions and future work is presented in Section 5.

## 2. Preliminaries

### 2.1. Notation

The space of real numbers is denoted by *ℜ*, and ${ℜ}^{k}$ denotes the *k*-dimensional Euclidean space with the Euclidean norm defined as $∥x∥=\sqrt{x^{⊤}x}$$\forall x\in {ℜ}^{k}$ where ${\left(\cdot \right)}^{⊤}$ is the transpose operator. The Special Orthogonal group of order 3 is denoted by

$$SO\left(3\right)=\{R\in {ℜ}^{3\times 3}\;|\;R^{⊤}R=I_{3},\det\left(R\right)=+1\}$$

where $I_{3}$ is the 3×3 identity matrix. The unit sphere embedded in ${ℜ}^{3}$ is defined as

$$S^{2}=\left\{x\in {ℜ}^{3}\;|\;x^{⊤}x=1\right\}.$$

### 2.2. Kinematic and Dynamics

**Figure 1:**
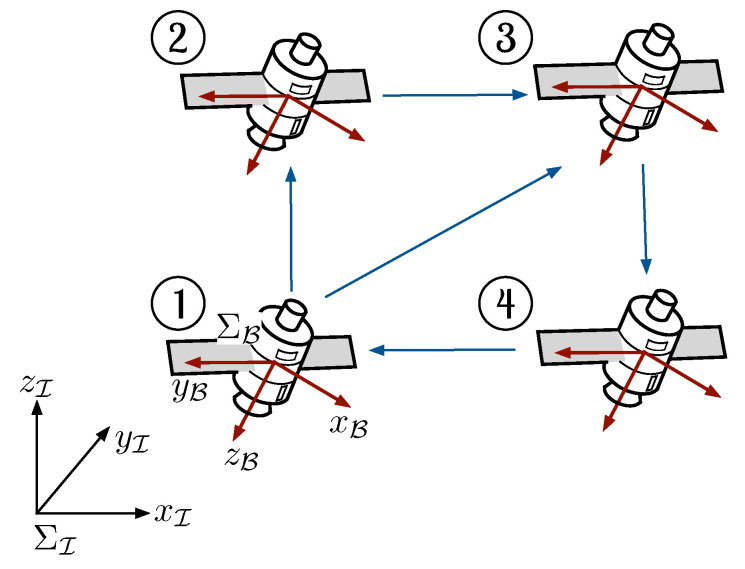
Swarm of rigid bodies with four elements. The rigid body’s attitude is obtained by projecting the body frame’s axes ($\Sigma_{B}$) with the inertia frame’s axes ($\Sigma_{I}$).

This section introduces the kinematics and dynamics models of a fully actuated rigid body. To begin with, let $\Sigma_{I}=\left\{x_{I},y_{I},z_{I}\right\}$ and $\Sigma_{B}=\left\{x_{B},y_{B},z_{B}\right\}$ denote the inertial and body frames, respectively (see Figure 1). The frames $\Sigma_{I}$ and $\Sigma_{B}$ are related by the rotation matrix R∈SO(3), which describes the attitude of the rigid body. The kinematics of the rigid body is given by [26]

(1) $$\begin{array}{c} \dot{R}=RS\left(\omega \right) \end{array}$$

where $\omega \in {ℜ}^{3}$ is the angular velocity resolved in the body frame $\Sigma_{B}$ and $S\left(\cdot \right)\in {ℜ}^{3\times 3}$ is a skew-symmetric matrix

(2) $$\begin{array}{c} S\left(a\right)=\left[\begin{array}{ccc} 0 & -a_{3} & a_{2}\\ a_{3} & 0 & -a_{1}\\ -a_{2} & a_{1} & 0 \end{array}\right],\forall a\in {ℜ}^{3}. \end{array}$$

**Figure 2:**
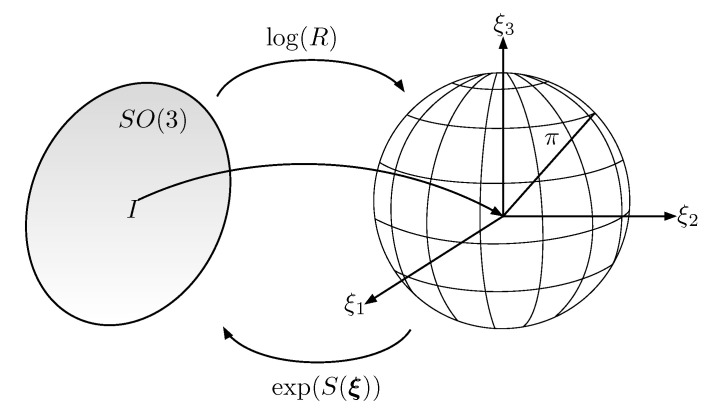
Relation between the exponential coordinates ξ and the rotation matrix R∈SO(3). The identity element $I_{3}$ on SO(3) is mapped to the origin ξ=0 in the exponential coordinates.

Alternatively to rotation matrices, the rigid body’s attitude can be described by the vector quantity $\xi =\theta n\in {ℜ}^{3}$ where $n\in S^{2}$ is the axis of rotation and θ∈ℜ is the rotation angle. The vector ξ is called the *exponentialcoordinates* of *R* and represents a minimal parametrization of the rigid body’s attitude [27]. The rotation matrix *R* and the exponential coordinates ξ are related by the exponential map

(3) $$\begin{array}{cc} R & =\exp(S(\xi ))\\ & =I_{3}+\frac{\sin\theta }{\theta }S\left(\xi \right)+\frac{1-\cos\theta }{\theta^{2}}S^{2}\left(\xi \right). \end{array}$$

where $I_{3}\in {ℜ}^{3\times 3}$ is the identity matrix and θ=∥ξ∥. Conversely, given R∈SO(3), we can compute S(ξ) by means of the logarithmic map defined as

(4) $$\begin{array}{c} \log\left(R\right)=S\left(\xi \right)=\frac{\theta }{\sin\theta }\left(\frac{R-R^{⊤}}{2}\right) \end{array}$$

where cosθ=(trace(R)−1)/2 and $(R-R^{⊤})/2$ is the skew-symmetric part of *R*. Finally, ξ is obtained as ξ=vec(log(R)) where vec(·) is the inverse of the matrix operator S(·), i.e., vec(S(a))=a for all $a\in {ℜ}^{3}$. The logarithmic operator (4) maps an element of SO(3) into a vector which is contained in a sphere of radius π and centered at the origin (see Figure 2). The points in the boundary of the sphere are the singularities of (4), i.e., when θ=π, or equivalently, when trace(R)=−1. It is important to remark that the set D={R∈SO(3)|trace(R)=−1} has zero measure; therefore, the exponential coordinates almost cover any rigid body’s attitude [28]. This is an important advantage with respect to other attitude parametrization such as Euler angles.

The kinematics of the exponential coordinates is given by [29]

(5) $$\begin{array}{c} \dot{\xi }=J\left(\xi \right)\omega \end{array}$$

where the Jacobian matrix $J\left(\xi \right)\in {ℜ}^{3\times 3}$ is given by

(6) $$\begin{array}{c} J\left(\xi \right)=I_{3}+\frac{1}{2}S\left(\xi \right)+\left(\frac{1}{\theta^{2}}-\frac{1+\cos\theta }{2\theta \sin\theta }\right)S^{2}\left(\xi \right) \end{array}$$

and its inverse is given by

(7) $$\begin{array}{c} J^{-1}\left(\xi \right)=I_{3}-\frac{1-\cos\theta }{\theta^{2}}S\left(\xi \right)+\frac{\theta -\sin\theta }{\theta^{3}}S^{2}\left(\xi \right). \end{array}$$

Notice that if ξ=0; then, $J\left(\xi \right)=J^{-1}\left(\xi \right)=I_{3}$. Morevoer, the Jacobian matrix J(ξ) and its inverse are well-defined for all ∥ξ∥<2π. The time derivatives of J(ξ) and $J^{-1}\left(\xi \right)$ are, respectively, given by

$$\begin{array}{cc} \dot{J}\left(\xi \right)= & \left(\frac{1}{\theta^{2}}-\frac{1+\cos\theta }{2\theta \sin\theta }\right)\left(S\left(\xi \right)S\left(\dot{\xi }\right)+S\left(\dot{\xi }\right)S\left(\xi \right)\right)+ \end{array}$$

(8) $$\begin{array}{cc} \; & \frac{1}{2}S\left(\dot{\xi }\right)+\left(\frac{\theta +\sin\theta }{2\theta^{2}\left(1-\cos\theta \right)}-\frac{2}{\theta^{3}}\right)\dot{\theta }S^{2}\left(\xi \right) \end{array}$$

(9) $$\begin{array}{cc} \frac{d}{dt}J^{-1}\left(\xi \right)= & -J^{-1}\left(\xi \right)\dot{J}\left(\xi \right)J^{-1}\left(\xi \right). \end{array}$$

where $\dot{\theta }=\xi^{⊤}\dot{\xi }/\theta$.

On the other hand, the equation of motion of a rigid body can be described by the Newton–Euler formalism [30],

(10) $$\begin{array}{c} M\dot{\omega }=\tau -S\left(\omega \right)M\omega \end{array}$$

where $M=M^{⊤}\in {ℜ}^{3\times 3}$ is the constant inertia matrix and $\tau \in {ℜ}^{3}$ is the input torque, both quantities are expressed in the body frame.

### 2.3. Graph Theory

We use tools of graph theory to model the communication between the rigid bodies. A graph G is composed by the set N={1,…,N} which contains the nodes (rigid bodies) and the set of edges (links) denoted E⊆N×N. For a directed graph or digraph, the edge set E is composed of ordered pairs of nodes; namely, the edge (i,j)∈E implies that the robot *i* obtains information from *j* but not vice versa. The adjacency matrix $A=\left[a_{ij}\right]\in {ℜ}^{N\times N}$ for a directed graph is defined as

(11) $$\begin{array}{c} a_{ij}=\left\{\begin{array}{cc} 1 & \mathrm{if}j\in N_{i}\\ 0 & \mathrm{otherwise} \end{array}\right. \end{array}$$

where $N_{i}$ is the set of neighbors transmitting information to rigid body *i*. The Laplacian matrix $L=\left[\ell_{ij}\right]\in {ℜ}^{N\times N}$ is defined as

(12) $$\begin{array}{c} \ell_{ij}=\left\{\begin{array}{cc} \sum_{k=1}^{N}a_{ik} & i=j\\ -a_{ij} & i\neq j. \end{array}\right. \end{array}$$

The Laplacian matrix has the eigenvector $1_{N}={\left[\begin{array}{ccc} 1 & \cdots & 1 \end{array}\right]}^{⊤}\in {ℜ}^{N}$ associated to the eigenvalue $\lambda_{1}=0$. For a directed graph, the rest of the spectrum of *L* has a positive real part. If there exists a sequence of edges (undirected path) that joins any pair of node, we say that the graph is connected [31]. A digraph is strongly connected if there is a directed path that connects every pair of nodes. If the eigenvalue $\lambda_{1}=0$ has an algebraic multiplicity of one, then the graph is connected [32,33,34].

## 3. Attitude Synchronization

Consider *N* rigid bodies with the state variables $\left(\xi_{i},\omega_{i}\right)$ and equation of motion described by

(13) $$\begin{array}{cc} {\dot{\xi }}_{i} & =J_{i}\left(\xi_{i}\right)\omega_{i} \end{array}$$

(14) $$\begin{array}{cc} M_{i}{\dot{\omega }}_{i} & =\tau_{i}-S\left(\omega_{i}\right)M_{i}\omega_{i} \end{array}$$

with i=1,…,N. Let

$$\tau_{i}=S\left(\omega_{i}\right)M_{i}\omega_{i}+M_{i}\left(u_{i}+{\dot{\omega }}_{i}^{r}\right)$$

where $u_{i}\in {ℜ}^{3}$ is an auxiliary control input and $\omega_{i}^{r}\in {ℜ}^{3}$ is an angular reference velocity that will be defined later. Based on the definitions given above, Equations (13) and (14) can be written as follows

(15) $$\begin{array}{cc} {\dot{\xi }}_{i} & =J_{i}\left(\xi_{i}\right)\omega_{i}^{r}+J_{i}\left(\xi_{i}\right){\tilde{\omega }}_{i} \end{array}$$

(16) $$\begin{array}{cc} {\dot{\tilde{\omega }}}_{i} & =u_{i} \end{array}$$

where ${\tilde{\omega }}_{i}=\omega_{i}-\omega_{i}^{r}\in {ℜ}^{3}$ is the angular velocity error.

In this work, the attitude synchronization problem can be stated as follows: design the reference velocity $\omega_{i}^{r}$ and the auxiliary control input $u_{i}$ such that

(17) $$\begin{array}{c} \lim_{t\to \infty }\xi_{i}\left(t\right)=\xi_{d}\left(t\right),\;\lim_{t\to \infty }\omega_{i}\left(t\right)=\omega_{d}\left(t\right),\;\forall i=1,\ldots ,N \end{array}$$

where $\xi_{d}\left(t\right)\in {ℜ}^{3}$ is the desired exponential coordinates which corresponds to the desired attitude $R_{d}\left(t\right)\in SO\left(3\right)$, namely, $\xi_{d}\left(t\right)=\mathrm{vec}\left(\log\left(R_{d}\left(t\right)\right)\right)$ and $\omega_{d}\left(t\right)\in {ℜ}^{3}$ is the desired angular velocity. The desired attitude satisfies the following kinematic equations

$${\dot{R}}_{d}=R_{d}S\left(\omega_{d}\left(t\right)\right),\;{\dot{\xi }}_{d}=J\left(\xi_{d}\right)\omega_{d}\left(t\right).$$

The proposed attitude synchronization control law is given by

(18) $$\begin{array}{cc} \omega_{i}^{r} & =J_{i}^{-1}\left(\xi_{i}\right)J\left(\xi_{d}\right)\omega_{d}\left(t\right)-k_{i}J_{i}^{-1}\left(\xi_{i}\right)\varphi_{i} \end{array}$$

(19) $$\begin{array}{cc} {\dot{\varphi }}_{i} & =-2k_{i}\varphi_{i}+k_{i}{\tilde{\xi }}_{i}+c\sum_{j=1}^{N}a_{ij}\left(\xi_{i}-\varphi_{i}\right)-\left(\xi_{j}-\varphi_{j}\right) \end{array}$$

(20) $$\begin{array}{cc} u_{i} & =-\gamma_{i}{\tilde{\omega }}_{i}-\alpha J_{i}^{⊤}\left(\xi_{i}\right)\left({\tilde{\xi }}_{i}-\varphi_{i}\right) \end{array}$$

where ${\tilde{\xi }}_{i}=\xi_{i}-\xi_{d}\left(t\right)\in {ℜ}^{3}$ is the attitude error in exponential coordinates, $\varphi_{i}\in {ℜ}^{3}$ is an additional state, $k_{i}$, $\gamma_{i}$, and α∈ℜ are positive constants, c∈ℜ is the positive coupling strength, and $a_{ij}$ denotes the elements of the adjacency matrix *A*. The time derivate of the reference angular velocity is given by

(21) $$\begin{array}{c} {\dot{\omega }}_{i}^{r}=J_{i}^{-1}\left(\xi_{i}\right)\left(J\left(\xi_{d}\right){\dot{\omega }}_{d}\left(t\right)+\dot{J}\left(\xi_{d}\right)\omega_{d}\left(t\right)-k_{i}{\dot{\varphi }}_{i}\right)+\left[\frac{d}{dt}J_{i}^{-1}\left(\xi_{i}\right)\right]\left(J\left(\xi_{d}\right)\omega_{d}-k_{i}\varphi_{i}\right). \end{array}$$

Notice that the time derivative of the angular velocity $\omega_{i}^{r}$ does not depend on the angular velocities of the other rigid bodies.

The following proposition summarizes the main contribution of this paper.

**Proposition**  **1.** *Consider a group of N rigid bodies described by (13) and (14) and assume that the communication graph G is directed and connected. Then, the dynamic control law (18)–(20) in closed loop with (15) and (16) achieves attitude synchronization in the sense of (17).*

**Proof.**  Substituting the synchronization control law (18)–(20) in (15) and (16) yields

(22) $$\begin{array}{cc} {\dot{\varphi }}_{i} & =-2k_{i}\varphi_{i}+k_{i}{\tilde{\xi }}_{i}+c\sum_{j=1}^{N}a_{ij}\left({\tilde{\xi }}_{i}-\varphi_{i}\right)-\left({\tilde{\xi }}_{j}-\varphi_{j}\right) \end{array}$$

(23) $$\begin{array}{cc} {\dot{\tilde{\xi }}}_{i} & =-k_{i}\varphi_{i}+J_{i}\left(\xi_{i}\right){\tilde{\omega }}_{i} \end{array}$$

(24) $$\begin{array}{cc} {\dot{\tilde{\omega }}}_{i} & =-\gamma_{i}{\tilde{\omega }}_{i}-\alpha J_{i}^{⊤}\left(\xi_{i}\right)\left({\tilde{\xi }}_{i}-\varphi_{i}\right) \end{array}$$

where ${\dot{\tilde{\xi }}}_{i}={\dot{\xi }}_{i}-J\left(\xi_{d}\right)\omega_{d}$ and $\left(\xi_{i}-\varphi_{i}\right)-\left(\xi_{j}-\varphi_{j}\right)=\left({\tilde{\xi }}_{i}-\varphi_{i}\right)-\left({\tilde{\xi }}_{j}-\varphi_{j}\right)$ have been used. To proceed with the stability analysis, we introduce the auxiliary state

(25) $$\begin{array}{c} r_{i}={\tilde{\xi }}_{i}-\varphi_{i} \end{array}$$

whose time derivative is given by

(26) $$\begin{array}{c} {\dot{r}}_{i}=-k_{i}r_{i}-c\sum_{j=1}^{N}a_{ij}\left(r_{i}-r_{j}\right)+J_{i}\left(\xi_{i}\right){\tilde{\omega }}_{i}. \end{array}$$

Finally, using the properties of the Kronecker product [35] and by taking into account (22)–(24) and (26), the closed-loop dynamics reads

(27) $$\begin{array}{cc} \dot{\varphi } & =-\left(K⊗I_{3}\right)\varphi +\left(\left(K+L\right)⊗I_{3}\right)r \end{array}$$

(28) $$\begin{array}{cc} \dot{r} & =-\left(\left(K+L\right)⊗I_{3}\right)r+J\left(\xi \right)\tilde{\omega } \end{array}$$

(29) $$\begin{array}{cc} \dot{\tilde{\omega }} & =-\left(\Gamma ⊗I_{3}\right)\tilde{\omega }-\alpha J^{⊤}\left(\xi \right)r \end{array}$$

where ⊗ denotes the Kronecker product, φ, ξ and $\tilde{\omega }\in {ℜ}^{3N}$ are stacked vectors, $L\in {ℜ}^{N\times N}$ is the Laplacian matrix, $J\left(\xi \right)=\mathrm{blockdiag}\left\{J_{1}\left(\xi_{1},\cdots ,J_{N}\left(\xi_{N}\right)\right)\right\}\in {ℜ}^{3N\times 3N}$ is the block diagonal matrix and $K=\mathrm{diag}\left\{k_{1},\cdots ,k_{N}\right\}\in {ℜ}^{N\times N}$, $\Gamma =\mathrm{diag}\left\{\gamma_{1},\ldots ,\gamma_{N}\right\}\in {ℜ}^{N\times N}$. Since the communication graph is connected by assumption and the matrix *K* is positive definite, the spectrum of K+L has a positive real part (see [36] for further details), and hence, the matrix $-\left(K+L\right)⊗I_{3}$ is Hurwitz. Therefore, it can be shown that the origin $\left(\varphi ,r,\tilde{\omega }\right)=\left(0,0,0\right)$ is the unique equilibrium point of (27)–(29). To analyze the stability of the equilibrium point, consider the canidate Lyapunov function

(30) $$\begin{array}{c} V=\frac{1}{2}\left(\mu \varphi^{⊤}\varphi +\alpha r^{⊤}r+{\tilde{\omega }}^{⊤}\tilde{\omega }\right) \end{array}$$

where μ∈ℜ is a positive constant. The candidate Lyapunov function can be lower and upper bounded as

(31) $$\begin{array}{c} a_{1}{∥x∥}^{2}\leq V\leq a_{2}{∥x∥}^{2} \end{array}$$

where $x={\left[\begin{array}{ccc} \varphi^{⊤} & r^{⊤} & {\tilde{\omega }}^{⊤} \end{array}\right]}^{⊤}\in {ℜ}^{9N}$ and $a_{1}=\frac{1}{2}\min\left\{\mu ,\alpha ,1\right\}\;$$a_{2}=\frac{1}{2}\max\left\{\mu ,\alpha ,1\right\}$. The time derivative of *V* along (27)–(29) is given by

(32) $$\begin{array}{cc} \dot{V}= & -\mu \varphi^{⊤}\left(K⊗I_{3}\right)\varphi +\mu \varphi^{⊤}\left(\left(K+L\right)⊗I_{3}\right)r-\alpha r^{⊤}\left(\left(K+L\right)⊗I_{3}\right)r\\ \; & -{\tilde{\omega }}^{⊤}\left(\Gamma ⊗I_{3}\right)\tilde{\omega }+\alpha r^{⊤}J\left(\xi \right)\tilde{\omega }-\alpha \tilde{\omega }J^{⊤}\left(\xi \right)r \end{array}$$

where the last two terms cancel each other out. The time derivate of *V* satisfies

(33) $$\begin{array}{cc} \dot{V} & \leq -{\mu k∥\varphi ∥}^{2}+\mu \lambda_{\max}\left\{K+L\right\}∥\varphi ∥∥r∥-\alpha \lambda_{\min}{\left\{K+L\right\}∥r∥}^{2}-\gamma {∥\tilde{\omega }∥}^{2}\\ \; & =-{\left[\begin{array}{c} ∥\varphi ∥\\ ∥r∥ \end{array}\right]}^{⊤}\underset{Q}{\underset{︸}{\left[\begin{array}{cc} \mu k & -\frac{1}{2}\mu \lambda_{\max}\left\{K+L\right\}\\ -\frac{1}{2}\mu \lambda_{\max}\left\{K+L\right\} & \alpha \lambda_{\min}\left\{K+L\right\} \end{array}\right]}}\left[\begin{array}{c} ∥\varphi ∥\\ ∥r∥ \end{array}\right]-\gamma {∥\tilde{\omega }∥}^{2} \end{array}$$

where $k=\min\{k_{1},\ldots ,k_{N}\}$, $\gamma =\min\{\gamma_{1},\ldots ,\gamma_{N}\}$. By selecting the parameter μ as

(34) $$\begin{array}{c} 0<\mu <\frac{4k\alpha \lambda_{\min}\left\{K+L\right\}}{\lambda_{\max}^{2}\left\{K+L\right\}} \end{array}$$

the symmetric matrix $Q\in {ℜ}^{2\times 2}$ is positive definite and hence $\dot{V}$ is a negative definite function. This in turn implies that the closed-loop variables are bounded and the origin is asymptotically stable in the sense of Lyapunov. It is important to remark that μ is not a controller parameter, and it can be made arbitrarily small to satisfy (34). By taking into account (31) and (34), it follows

(35) $$\begin{array}{c} \dot{V}\leq -a_{3}{∥x∥}^{2}\leq -\frac{a_{3}}{a_{2}}V\Rightarrow V\left(t\right)\leq V\left(0\right)\exp\left(-\frac{a_{3}}{a_{2}}t\right). \end{array}$$

where $a_{3}=\min\left\{\lambda_{\min}\left\{Q\right\},\gamma \right\}$. The inequality (35) implies that the origin $\left(\varphi ,r,\tilde{\omega }\right)=\left(0,0,0\right)$ is also an exponentially stable equilibrium point [37,38]. Since the exponential coordinates almost cover any rotation in SO(3), the domain of attraction of the equilibrium point is almost global. The exponential convergence to zero of r(t) and φ(t) implies

$$\lim_{t\to \infty }\tilde{\xi }\left(t\right)=0\Rightarrow \lim_{t\to \infty }\xi_{i}\left(t\right)=\xi_{d}\left(t\right)\;\forall i\in N.$$

The exponential converge of $\tilde{\omega }\left(t\right)$ to zero also implies that

$$\lim_{t\to \infty }\omega_{i}\left(t\right)=\omega_{i}^{r}\left(t\right)=J_{i}^{-1}\left(\xi_{i}\right)J\left(\xi_{d}\right)\omega_{d}-k_{i}J\left(\xi_{i}\right)\varphi_{i}.$$

Since φ(t)→0 and $\xi_{i}\left(t\right)\to \xi_{d}\left(t\right)$ as t→∞, it follows

$$\lim_{t\to \infty }\omega_{i}\left(t\right)=\omega_{d}\left(t\right).$$

This concludes the proof. □

## 4. Simulations

This section presents simulation results to show the performance of the synchronization control law developed in Section 3. In the simulation, we consider a network of four rigid bodies whose inertia matrices are given by

$$\begin{array}{cc} M_{1}=M_{3} & =\left[\begin{array}{ccc} 1.5 & 0.25 & 0.25\\ 0.25 & 2 & 0.4\\ 0.25 & 0.4 & 1.75 \end{array}\right]\left[{\mathrm{Kgm}}^{2}\right]\\ M_{2}=M_{4} & =\left[\begin{array}{ccc} 3.2 & 0.11 & -0.03\\ 0.11 & 3 & 0.08\\ -0.03 & 0.08 & 3.1 \end{array}\right]\left[{\mathrm{Kgm}}^{2}\right]. \end{array}$$

From the Rodrigues’ rotation formula, the attitude of the four rigid bodies can be expressed as

(36) $$\begin{array}{c} R_{i}=n_{i}n_{i}^{⊤}+\cos\theta_{i}\left(I_{3}-n_{i}n_{i}^{⊤}\right)+\sin\theta_{i}S\left(n_{i}\right). \end{array}$$

We recall that $\left(\theta_{i},n_{i}\right)$ are the angle/axis of rotation. The initial attitude as a function of the angle/axis parameters is described in Table 1. The four rigid bodies start at the rest position; this implies that the initial angular velocity is zero, i.e., $\omega_{i}\left(0\right)=0\in {ℜ}^{3}$ [rad/s]. In the simulation, the control gains were selected as

$$K=2I_{4},\;\Gamma =I_{4},\;c=2,\;\alpha =1$$

where $I_{4}$ is a 4×4 identity matrix. The communication topology is depicted in Figure 1, and its corresponding Laplacian matrix is given by

$$L=\left[\begin{array}{cccc} 1 & 0 & 0 & -1\\ -1 & 1 & 0 & 0\\ -1 & -1 & 2 & 0\\ 0 & 0 & -1 & 1 \end{array}\right].$$

The graph is connected and the spectrum of *L* is given by $\sigma \left\{L\right\}=\left\{0,1.5\pm \sqrt{3}/2j,2\right\}$. The desired angular velocity and its time derivative are given by

$$\begin{array}{cc} \omega_{d}\left(t\right) & =\frac{1}{4}{\left[\begin{array}{ccc} \sin(t) & 0 & \cos(t) \end{array}\right]}^{⊤}\left[\mathrm{rad}/s\right]\\ {\dot{\omega }}_{d}\left(t\right) & =\frac{1}{4}{\left[\begin{array}{ccc} \cos(t) & 0 & -\sin(t) \end{array}\right]}^{⊤}\left[\mathrm{rad}/s^{2}\right]. \end{array}$$

The desired exponential coordinates are given by $\xi_{d}\left(t\right)=\mathrm{vex}\left(R_{d}\left(t\right)\right)$ where $R_{d}\left(t\right)$ is the solution of ${\dot{R}}_{d}=R_{d}S\left(\omega_{d}\left(t\right)\right)$.

In order to perform the simulation, the kinematic and dynamic models of the rigid body were discretized as follows

$$\begin{array}{cc} R_{k+1} & =R_{k}\exp\left(S(T\omega_{k})\right)\\ \omega_{k+1} & =\omega_{k}+TM^{-1}\left(\tau_{k}-S\left(\omega_{k}\right)M\omega_{k}\right) \end{array}$$

where $R_{k}=R\left(kT\right)$, $\omega_{k}=\omega \left(kT\right)$, $\tau_{k}=\tau \left(kT\right)$, and T=0.01 [ms] is the time step size with k=0,1,2,…,N. Since the matrix exponential $\exp\left(S(T\omega_{k})\right)$ is in fact a rotation matrix (see Equation (3)), at each integration step, the structure and properties of $R_{k}$ are preserved. The desired attitude $R_{d}$ is computed in a similar fashion, i.e.,

$$R_{d}\left(\left(k+1\right)T\right)=R_{d}\left(kT\right)\exp\left(S\left(\omega_{d}\left(kT\right)\right)\right),\;R_{d}\left(0\right)=I_{3}.$$

**Figure 3:**
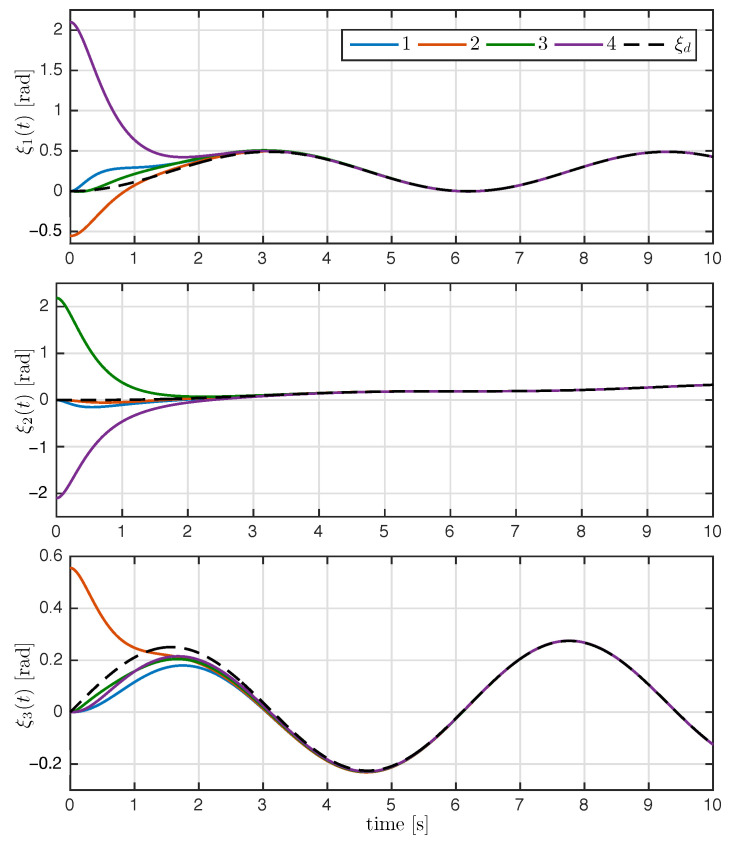
Time evolution of the exponential coordinates $\xi_{i}\left(t\right)$ with i=1,2,3,4; the dashed line denotes the desired attitude $\xi_{d}\left(t\right)$.

**Figure 4:**
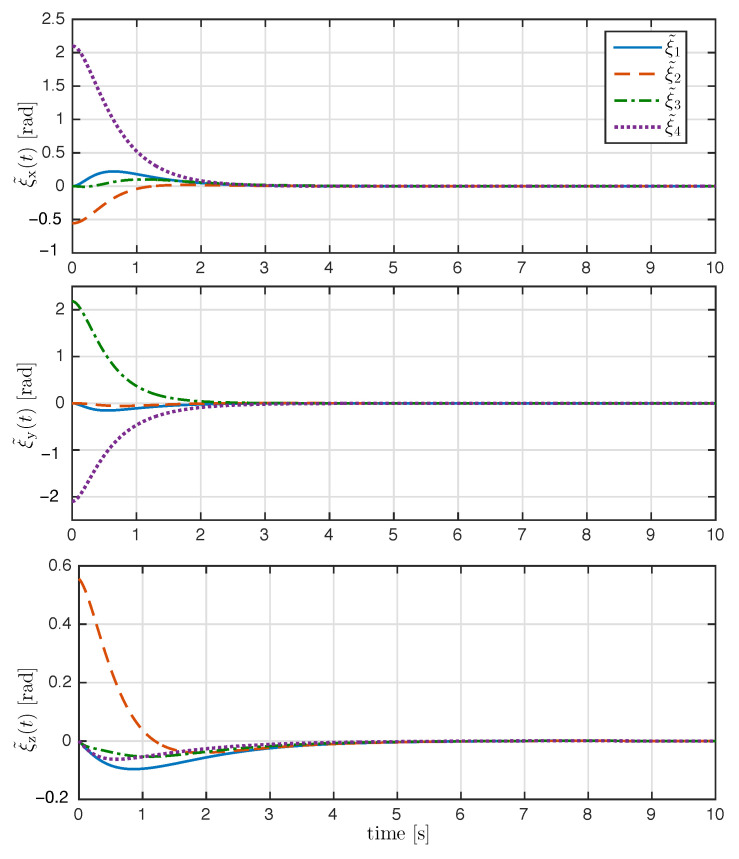
Time evolution of the attitude tracking error ${\tilde{\xi }}_{i}\left(t\right)$ (rad) with i=1,2,3,4.

**Figure 5:**
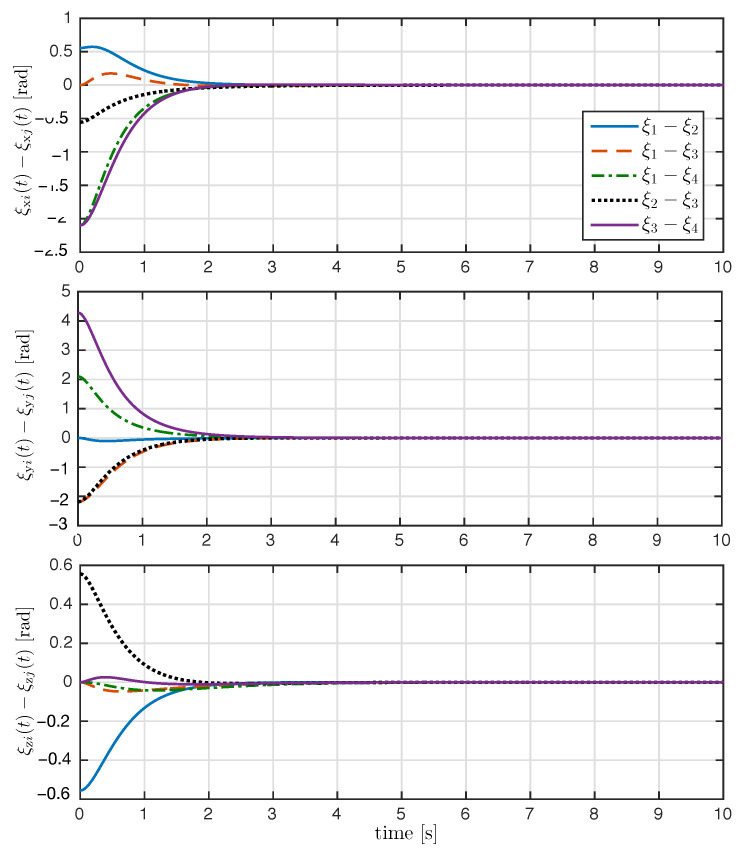
Time evolution of the relative attitude error $\xi_{i}\left(t\right)-\xi_{j}\left(t\right)$ (rad) for all i,j∈N.

The trajectory of the exponential coordinates is shown in Figure 3. As seen in Figure 3, the proposed control law achieved attitude synchronization; after the transient response, the exponential coordinates of each rigid body converge to the desired attitude $\xi_{d}\left(t\right)$. The time evolution of the attitude error ${\tilde{\xi }}_{i}\left(t\right)$ and relative attitude error $\xi_{i}\left(t\right)-\xi_{j}\left(t\right)$ for all i,j∈N are shown in Figure 4 and Figure 5, respectively. As it can be appreciated in both figures, the attitude errors converge exponentially to zero with a very similar convergence rate.

**Figure 6:**
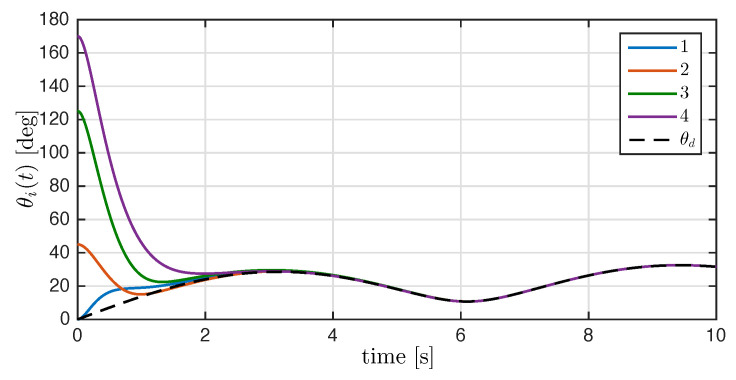
Time evolution of the rotation angle $\theta_{i}\left(t\right)$ with i=1,2,3,4, and the dashed line denotes the desired rotation angle $\theta_{d}\left(t\right)$.

To obtain a better insight of the attitude synchronization, we compute the angle of rotation and desired angle as follows

$$\theta_{i}=\cos^{-1}\left(\frac{\mathrm{trace}(R_{i})-1}{2}\right),\;\theta_{d}\left(t\right)=\cos^{-1}\left(\frac{\mathrm{trace}(R_{d}\left(t\right))-1}{2}\right).$$

for all i∈N. The time evolution of the angle of rotation of each rigid body is depicted in Figure 6. After the transient response, the angles of rotation converge to the desired rotation angle $\theta_{d}\left(t\right)$.

**Figure 7:**
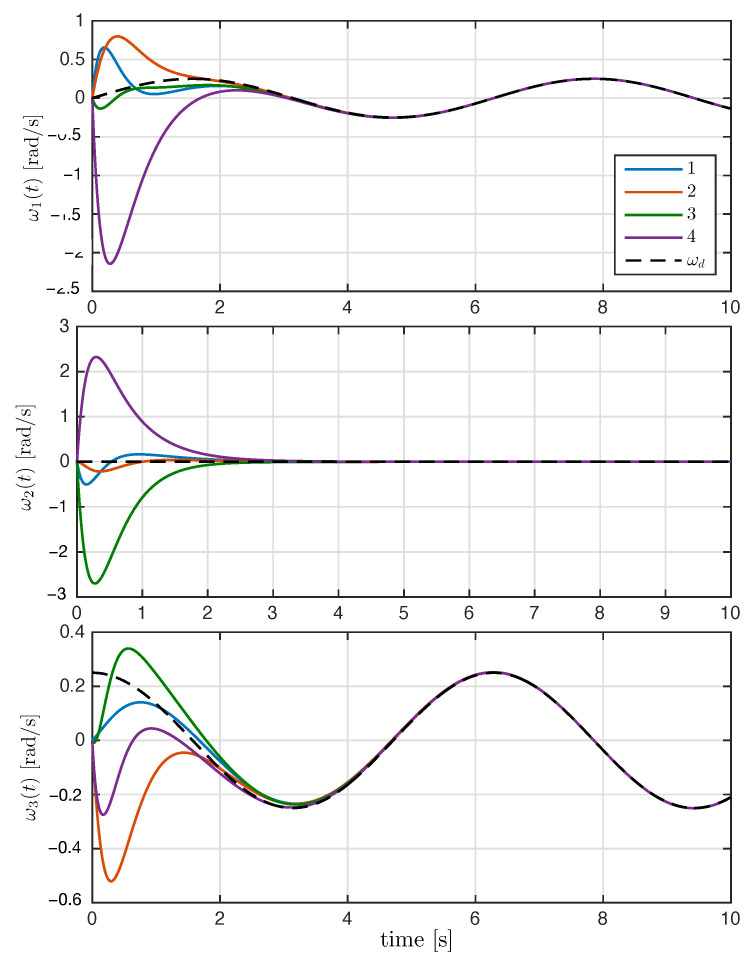
Time evolution of rigid bodies’ angular velocity $\omega_{i}\left(t\right)$ with i=1,2,3,4, and the dashed line denotes the desired angular velocity $\omega_{d}\left(t\right)$.

**Figure 8:**
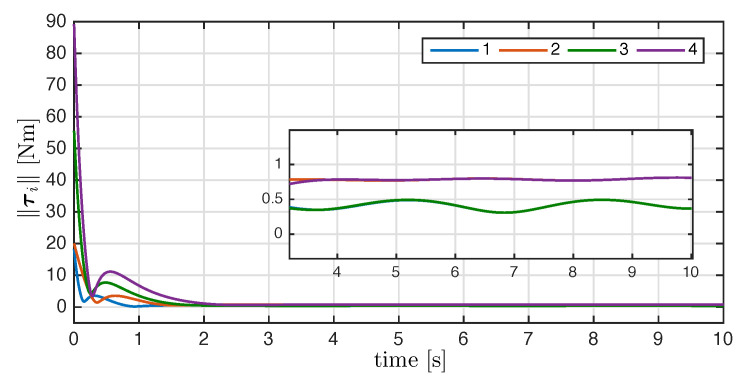
Norm of the control input $\tau_{i}\left(t\right)$ with i=1,2,3,4.

On the other hand, Figure 7 shows the components of the angular velocities for each agent, which converge exponentially to the desired angular velocity, meaning that the attitude synchronization objective is reached. Finally, the magnitude of the control input torque generated by the synchronization control law is shown in Figure 8.

## 5. Conclusions

In this work, we proposed a control law based on the exponential coordinates of rotation to solve the attitude synchronization problem of a group of fully actuated rigid bodies over directed communication topologies. To design the synchronization controller, we exploit the cascade structure between the rigid body’s kinematics and dynamics. In this regard, we divided the problem into an outer-loop controller (kinematic controller) and an inner-loop controller (velocity tracking controller). The former controller was designed to achieve attitude synchronization using a reference angular velocity as the virtual control input. On the other hand, for the latter controller, we designed a trajectory tracking controller that drives the angular velocity error to zero. The proposed controller for rigid body *i* achieves attitude synchronization using only its angular velocity and the attitude of its neighbors. The neighbors’ angular velocity is not required to achieve attitude synchronization. By means of a strict Lyapunov function, we showed that the equilibrium point of the closed-loop dynamics is exponentially stable. Numerical simulation shows the effectiveness of the proposed approach. As future work, we will consider communication delays and parameter uncertainties in the dynamic model.

## Figures and Tables

**Table 1:** Initial orientation of the four rigid bodies.

Index	1	2	3	4
$\theta_{i}\left(0\right)$	0°	45°	125°	170°
$n_{i}\left(0\right)$	arbitrary	$\frac{1}{\sqrt{2}}\left[\begin{array}{c} -1\\ 0\\ 1 \end{array}\right]$	$\left[\begin{array}{c} 0\\ 1\\ 0 \end{array}\right]$	$\frac{1}{\sqrt{2}}\left[\begin{array}{c} 1\\ -1\\ 0 \end{array}\right]$
